# Predicting epidermal growth factor receptor mutations in non-small cell lung cancer through dual-layer spectral CT: a prospective study

**DOI:** 10.1186/s13244-024-01678-9

**Published:** 2024-04-29

**Authors:** Fenglan Li, Linlin Qi, Sainan Cheng, Jianing Liu, Jiaqi Chen, Shulei Cui, Shushan Dong, Jianwei Wang

**Affiliations:** 1 Department of Diagnostic Radiology, National Cancer Center/National Clinical Research Center for Cancer/Cancer Hospital, Chinese Academy of Medical Sciences and Peking Union Medical College, No. 17 Panjiayuan Nanli, Beijing, Chaoyang District 100021 China; 2 Clinical Science, Philips Healthcare, Beijing, China

**Keywords:** Carcinoma (non-small-cell lung), Lung neoplasms, Epidermal growth factor receptor, Spectral computed tomography

## Abstract

**Objective:**

To determine whether quantitative parameters of detector-derived dual-layer spectral computed tomography (DLCT) can reliably identify epidermal growth factor receptor (EGFR) mutation status in patients with non-small cell lung cancer (NSCLC).

**Methods:**

Patients with NSCLC who underwent arterial phase (AP) and venous phase (VP) DLCT between December 2021 and November 2022 were subdivided into the mutated and wild-type EGFR groups following EGFR mutation testing. Their baseline clinical data, conventional CT images, and spectral images were obtained. Iodine concentration (IC), iodine no water (INW), effective atomic number (Zeff), virtual monoenergetic images, the slope of the spectral attenuation curve (λ_HU_), enhancement degree (ED), arterial enhancement fraction (AEF), and normalized AEF (NAEF) were measured for each lesion.

**Results:**

Ninety-two patients (median age, 61 years, interquartile range [51, 67]; 33 men) were evaluated. The univariate analysis indicated that IC, normalized IC (NIC), INW and ED for the AP and VP, as well as Zeff and λ_HU_ for the VP were significantly associated with EGFR mutation status (all *p* < 0.05). INW(VP) showed the best diagnostic performance (AUC, 0.892 [95% confidence interval {CI}: 0.823, 0.960]). However, neither AEF (*p* = 0.156) nor NAEF (*p* = 0.567) showed significant differences between the two groups. The multivariate analysis showed that INW(AP) and NIC(VP) were significant predictors of EGFR mutation status, with the latter showing better performance (*p* = 0.029; AUC, 0.897 [95% CI: 0.816, 0.951] vs. 0.774 [95% CI: 0.675, 0.855]).

**Conclusion:**

Quantitative parameters of DLCT can help predict EGFR mutation status in patients with NSCLC.

**Critical relevance statement:**

Quantitative parameters of DLCT, especially NIC(VP), can help predict EGFR mutation status in patients with NSCLC, facilitating appropriate and individualized treatment for them.

**Key Points:**

- Determining EGFR mutation status in patients with NSCLC before starting therapy is essential.
- Quantitative parameters of DLCT can predict EGFR mutation status in NSCLC patients.
- NIC in venous phase is an important parameter to guide individualized treatment selection for NSCLC patients.

**Graphical Abstract:**

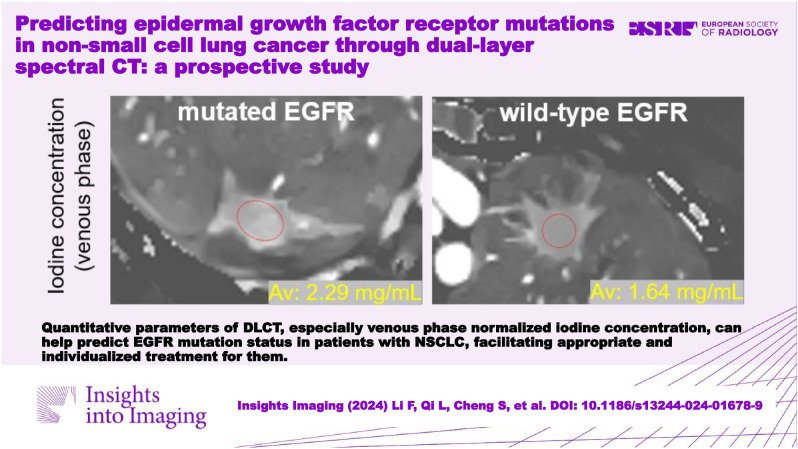

## Introduction

With an estimated 2.21 million new cases and 1.80 million deaths worldwide per year, lung cancer is one of the most frequently diagnosed cancers [1]. In the 2016 cancer statistics by the National Cancer Center, lung cancer ranked first both in the incidence (59.89%) and death rate (47.51%) of malignant tumors in China [2]. Non-small cell lung cancer (NSCLC) accounts for more than 80% of cases and is considered a heterogeneous disease [3]. With the discovery of the epidermal growth factor receptor (EGFR) gene and continuous research on tyrosine kinase inhibitor (TKI), treatment of advanced or metastatic patients with NSCLC has been greatly improved, and the overall survival has been extended [4, 5]. EGFR-TKI is the standard first-line treatment for NSCLC patients with EGFR mutations [6]. EGFR mutations associated with NSCLC mainly comprise exons 18 to 21, with a deletion in exon 19 and an L858R mutation in exon 21 (EGFR-sensitizing mutations) accounting for approximately 90% of them [7, 8]. Positive rates of EGFR mutations were as high as 41–48% in China [9, 10]. Thus, determining the EGFR mutation status of patients with NSCLC before starting EGFR-TKI therapy is crucial.

Gene mutational sequencing of tumor tissue from biopsy specimens is the gold standard for detecting EGFR mutations. However, obtaining tissue samples from the tumor is sometimes difficult owing to the tumor location and size, the potential risk of metastasis, and the relatively high costs [11, 12]. Therefore, exploring a non-invasive and readily available method to predict EGFR mutation status in patients with NSCLC is necessary.

Computed tomography (CT) features and certain CT-based radiomic features of lung cancer have recently been revealed to be related to EGFR mutation status [13–16]. Although CT image evaluation is not a substitute for tissue biopsy, it can provide information throughout the treatment to compensate for the lack of biopsy information. Furthermore, prediction of EGFR mutation status by CT imaging could help physicians select the most representative tumor for biopsy when multiple tumors are present. However, these CT features cannot be quantitatively evaluated owing to the subjective judgment of observers. Detector-derived dual-layer spectral CT (DLCT) provides various quantitative analysis tools and a comprehensive diagnostic model based on multi-parameter imaging, using a single X-ray source and two-layer detectors, with an upper layer absorbing low-energy photons and a lower layer absorbing high-energy photons [17]. The virtual non-contrast (VNC) image, virtual monochromatic image (VMI), iodine concentration (IC) image, iodine no water (INW) image, the slope of the spectral attenuation curves (λ_HU_), effective atomic number (Zeff) image, and normalized arterial enhancement fraction (NAEF) map can be generated along with the conventional CT images. Compared with dual-energy CT (DECT) in previous studies, DLCT can produce better data registration and image correspondence in different phases through its synchronization, homology, and co-direction features, and significantly reduce measurement errors, image noise, and tedious and repetitive image reconstruction or post-processing procedures [18]. Moreover, DECT has potential value in predicting EGFR mutation status in lung cancer, with AUCs ranging from 0.702 to 0.760 [19, 20]. However, this diagnostic performance is not optimal and is inconsistent owing to differences in equipment, scanning parameters, sample size, and pathological types. Consequently, the diagnostic value of DECT quantitative parameters should be further explored. In addition, the value of each DLCT quantitative parameter in predicting EGFR mutation status in NSCLC has not been reported.

Therefore, we aimed to prospectively explore the potential value of DLCT quantitative parameters in the identification of EGFR mutation status in NSCLC by strictly controlling the CT scanning phase and standardizing these parameters.

## Methods

### Study population

This prospective study was approved by the relevant institutional review board, and the requirement for informed consent was waived.

**Fig. 1:**
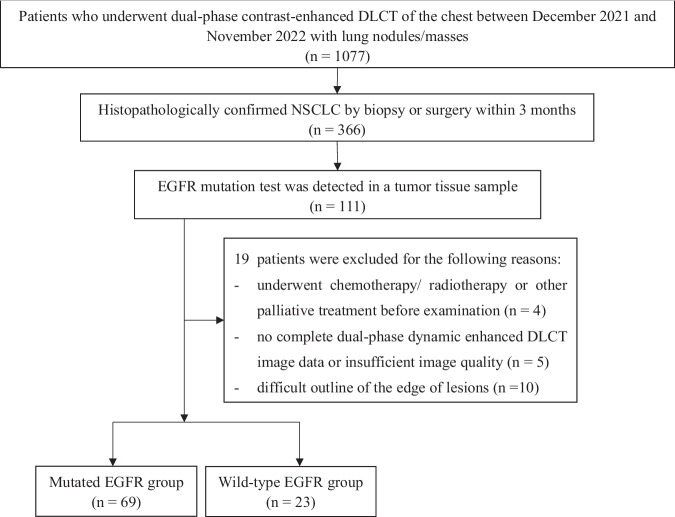
Flowchart demonstrates the study inclusion process prospectively undergone by patients with NSCLC and EGFR mutation test

Patients with lung nodule(s) or mass(es) who underwent chest DLCT examination in our hospital were prospectively enrolled from December 2021 to November 2022. The inclusion criteria were: (1) patients who underwent chest dual-phase dynamic enhanced scan (arterial phase [AP] and venous phase [VP]) with DLCT; (2) NSCLC confirmed by pathological examination after biopsy or surgical resection; (3) ≤ 3-month interval between the DLCT scan and surgery/biopsy examination; and (4) EGFR mutation testing. The exclusion criteria were: (1) patients with a history of chemical/radiotherapy treatment before the CT scan; (2) patients with incomplete imaging data, poor image quality, or respiratory artifacts; (3) tumor surroundings exhibiting atelectasis or patchy shadows that prevented accurate observation of details or lesion size measurement; (4) incomplete clinical data on the Management Information System of our hospital. According to EGFR mutation status, all patients were subdivided into the mutated and the wild-type EGFR groups (Fig. 1).

Finally, 92 patients with NSCLC (33 men and 59 women; median age, 61 years [interquartile range {IQR}, 51–67 years]) were included, with the mutated EGFR group featuring 69 patients (21 men and 48 women; median age, 61 years [IQR, 51–65 years]) and the wild-type EGFR group 23 patients (12 men and 11 women; mean age, 61.4 ± 11.0 years).

## DLCT acquisition and post-processing

All patients underwent DLCT (IQon Spectral CT; Philips Healthcare), followed by chest dual-phase dynamic enhanced scans because most of the patients were admitted to our hospital with pulmonary nodules or masses found by examination in other hospitals. Furthermore, some patients had a purely clinical diagnosis of lung cancer, and some had been diagnosed by biopsy. To further determine lung cancer stage, the scopes of preoperative CT scans for some patients were extensive and usually included abdomen, pelvic, neck, or head scans after the chest scan. Therefore, the scope of the scan was not consistent for each patient, but consistency in the timing of chest scans was ensured.

Before the scan, an anterior-posterior scout was performed to determine the scan range. Intravenous contrast medium (Imeron 400 MCT, 400 mg/mL; Bracco Imaging) was injected at a standard dosage (80–90 mL) at a flow rate of 3 mL/s using a high-pressure injector (Ulrich REF XD 2051, Ulrich GmbH & Co. KG), followed by a 30-mL saline chaser at the same flow rate. AP and VP images were acquired 35 and 65 s after the injection, respectively. Considering that lung cancer typically shows peak enhancement 20–40 s after initiating the injection, a 65 s fixed delay ensured analysis of the tumor during the late AP or the early phase of the steady decrease enhancement occurring after that. This delayed acquisition did not prevent the scanning of the upper abdomen at the portal phase. The following scanning parameters were used: 120 kVp; automatic tube current selection with resulting exposures of 37–84 mAs; rotation speed, 0.33 s/rot; helical pitch, 0.671; detector collimation, 64 × 0.625 mm; and 512 × 512 matrix. Images were reconstructed as spectral base images (SBI) datasets, with a reconstructed slice thickness of 1 mm, and an increment of 1 mm. Conventional CT images were reconstructed using hybrid iterative reconstruction (iDose 4, level 4, Philips Healthcare) and a standard kernel (B), reviewed in a mediastinal window with a width of 350 and a level of 40. The same protocols were applied to all participants.

### DLCT image analysis

Quantitative analysis of DLCT images using commercially available software (IntelliSpace Portal v. 10.1, Philips Healthcare) was performed in consensus by a resident fellow in radiology with three years of radiology experience and a senior radiologist with 30 years of radiology experience.

All lesions were observed and recorded on conventional CT images by the mediastinal window (width and level, 350 HU and 40 HU), lung window (width and level, 1600 HU and −600 HU), and multiplanar reconstruction (MPR) technology. When multiple lesions were present, only the largest was considered. According to the density features on the conventional CT images with a lung window, the target lesions were divided into three categories: solid, part-solid, and ground glass opacity (GGO) types. We recorded the location, size (diameter, maximum long-axis diameter, and maximum short-axis diameter perpendicular to maximum long-axis), and morphological features (lobulation, spiculation, bubble sign, and pleural retraction).

**Fig. 2:**
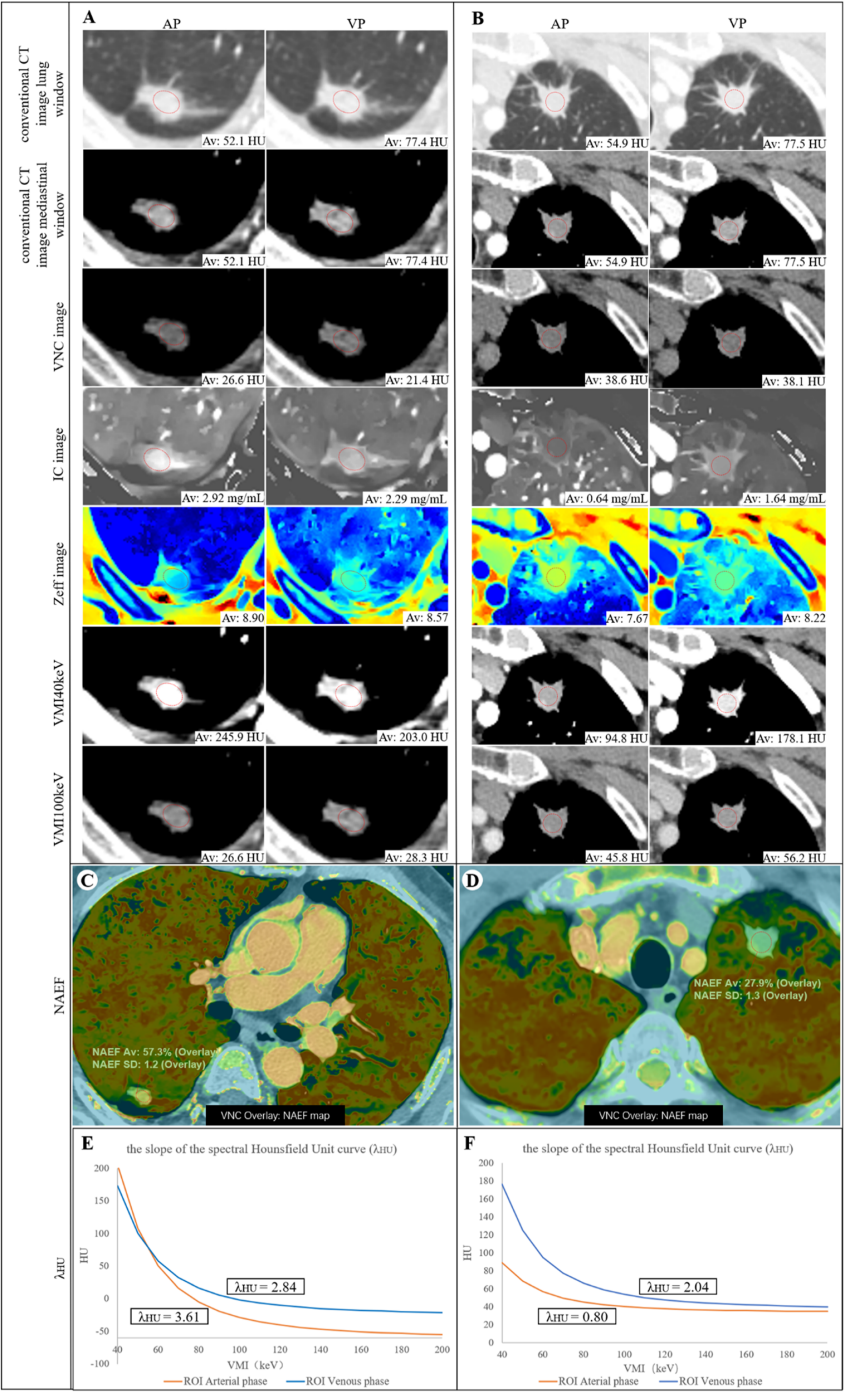
**A**, **C**, **E** Images of a 65-year-old man with lung adenocarcinoma in the right lower of the mutated epidermal growth factor receptor (EGFR) group. **B**, **D**, **F** Images of a 63-year-old woman with lung adenocarcinoma in the left upper lobe of the wild-type EGFR group. Conventional CT value and detector-derived dual-layer spectral CT parameters including virtual non-contrast (VNC), iodine concentration (IC), effective atomic number (Zeff), virtual monochromatic image (VMI) at 40 keV level (hereafter, VMI 40 keV), VMI at 100 keV level (hereafter, VMI 100 keV), normalized arterial enhancement fraction (NAEF), and the slope of the spectral Hounsfield Unit curve (λHU) at 40 keV–200 keV levels during the arterial phase (AP) and venous phase (VP) were measured with the same region of interest (ROI) at the same location

The circular or ovular region of interest (ROI) in the largest size level of the target lesion was manually placed in the conventional CT axial image in the AP, avoiding the vessels, calcification, necrosis and vacuoles/cavities inside the tumor. The ROI area covered at least half to two-thirds of the entire lesion. Subsequently, the ROI was manually replicated on the same site in the VP and automatically copied onto each spectral image of dual-phase, including IC, VMI at 40 keV and 100 keV (hereafter VMI_(40keV)_ and VMI_(100keV)_), VNC, Zeff, and INW. Similarly, circular ROIs were placed in the descending aorta at the carina level to obtain the IC of the aorta. Dual-phase DLCT data sets are registered on each other to precisely align them in three dimensions. NAEF was then calculated for each pixel, and data were mapped to a spectral color scale and overlaid with a VNC image (Fig. 2, Table 1).

**Table 1:** The quantitative parameters of detector-derived dual-layer spectral CT

Quantitative parameters (abbreviation, unit)	Specification	Characteristic
Virtual Monoenergetic Image (VMI, HU)	Virtually synthesize monoenergetic images, including 40 keV–200 keV.	The high energy level (80 keV–200 keV) can reduce the hardening beam effect and image artifacts; the low energy level (40 keV–60 keV) can increase the iodine contrast agent and enhance the visualization effect of the tissue.
Virtual non-contrast (VNC, HU)	Virtually remove the iodine element to obtain a virtual plain scan image.	Simplify the scanning process and reduce radiation dose.
Iodine no water (INW, mg/mL)	Suppression of watery tissue through substance identification to enhance visualization of iodine-enhanced tissue.	The iodine concentration of individual voxels can be displayed.
Iodine concentration (IC, mg/mL) Normalized iodine concentration (NIC, mg/mL)	Displays the iodine concentration of the tissue and standardized the IC by linking it to the artery.	Quantify iodine enhancement and display only the tissues containing iodine contrast agent.
Z Effective (Zeff)	The effective atomic number reconstructed through mass attenuation coefficients on material.	Perform material detection, identification and material separation.
The slope of the spectral attenuation curve (λ_HU_)	The slope of the curve of CT values changing with monochromatic energy.	Distinguish tissues based on the respective curve slopes
Arterial enhancement fraction (AEF, %) Normalized arterial enhancement fraction (NAEF, %)	The ratio of the IC or NIC during the arterial phase to the venous phase.	Reflecting the ratio of blood supply during the arterial and venous phases.

The related spectral parameter formula was as follows: normalized IC (NIC) = IC of the lung lesion/IC of the aorta; arterial enhancement fraction (AEF) = (IC in the AP/IC in the VP) × 100%; NAEF = (NIC in the AP/NIC in the VP) × 100%; enhancement degree (ED) = (conventional CT value − VNC). The software automatically calculated the value of Zeff and INW. For energy levels greater than 120 keV, the spectral curve exhibited smaller changes and differences compared to those below 120 keV. Therefore, VMI_(40keV)_ and VMI_(100keV)_ were selected for analysis, corresponding to λ_HU_ = (VMI_(40keV)_−VMI_(100keV)_)/(100−40).

### Statistical analysis

Statistical analyses were performed using the SPSS software (version 26.0; IBM Corporation) and MedCalc software (Version 20.121). Continuous data with normal distribution are expressed as mean ± standard deviation; otherwise, the medians with interquartile range (IQR, p25–p75) are presented. Categorical variables are expressed as numbers (percentage, %). Potentially significant factors for predicting EGFR mutation status in NSCLC were analyzed using univariate and multivariate analyses. In univariate analyses, Student’s *t*-test/Mann–Whitney U-test and the chi-square test/Fisher’s exact test were used to compare continuous and categorical variables between the groups, respectively. Factors with an associated *p* < 0.1 in the univariate analysis were selected as candidate variables to establish the multinomial logistic regression model, and the forward LR elimination was performed to determine the best independent predictor. Receiver operating characteristics curve analysis was performed, and AUCs were calculated to assess the predictive value of DLCT parameters. The threshold value with the maximum Youden index was chosen as optimal, and the sensitivity, specificity, and accuracy were calculated. The level of significance was set at *p* < 0.05.

## Results

### Participant characteristics

A total of 92 patients were eligible, including 87 (94.6%) cases of lung adenocarcinoma and 5 (5.4%) cases of lung squamous cell carcinoma. Five cases (5.4%) had a history of other malignancies (two of thyroid cancer, one of breast cancer, one of colon cancer, and one of prostate cancer). EGFR mutation types included mutation in exon 18 (2/69, 2.9%), exon 19 (25/69, 36.2%), exon 20 (3/69, 4.3%), exon 21 (38/69, 55.1%), and exons 18 and 21 (1/69, 1.4%). The association of clinical characteristics with EGFR mutation status is shown in Table 2. No significant differences were observed between the two groups for any clinical characteristic (*p* > 0.05). Candidate variables were age, sex, and smoking status.

**Table 2:** Study sample characteristics

Variables	All	Mutated EGFR group	Wild-type EGFR group	*p*
No. of patients	92	69 (75.0%)	23 (25.0%)	
Age (years)	61 (51, 67)	61 (51, 65)	61 ± 11	0.090
Sex ratio				0.060
Female	59 (64.1%)	48 (81.4%)	11 (18.6%)	
Male	33 (35.9%)	21 (63.6%)	12 (36.4%)	
History of malignancy				0.675
Not have	87 (94.6%)	65 (74.7%)	22 (24.3%)	
Have	5 (5.4%)	4 (80.0%)	1 (20.0%)	
Smoking				0.071
Never smoked	69 (75.0%)	55 (79.7%)	14 (20.3%)	
Smoker	23 (25.0%)	14 (60.9%)	9 (39.1%)	
Family history of malignant tumor				0.778
Not have	70 (76.1%)	52 (74.3%)	18 (25.7%)	
Have	22 (23.9%)	17 (77.3%)	5 (22.7%)	
TNM stage				0.733
I	62 (67.4%)	46 (74.2%)	16 (25.8%)	
II	8 (8.7%)	6 (75.0%)	2 (25.0%)	
III	6 (6.5%)	5 (83.3%)	1 (16.7%)	
IV	9 (9.8%)	8 (88.9%)	1 (11.1%)	
Indefinite	7 (7.6%)	4 (57.1%)	3 (42.9%)	

Unless otherwise indicated, data in parentheses are percentages; mean data are ± standard deviation; median data are interquartile range (p25–p75)

*EGFR* epidermal growth factor receptor

### Conventional CT image analysis

There were no significant differences between the mutated and wild-type EGFR groups in location, size, density, CT morphological characteristics of the target lesion, or number of lesions (*p* > 0.05; Table 3). The candidate variable was lesion lobulation.

**Table 3:** Association between conventional CT imaging features with EGFR mutation status in NSCLC

Variables	All	Mutated EGFR group	Wild-type EGFR group	*p*
Location				0.284
Right upper lobe	26 (28.3%)	21 (80.8%)	5 (19.2%)	
Right middle lobe	9 (9.8%)	8 (88.9%)	1 (11.1%)	
Right lower lobe	8 (8.7%)	4 (50.0%)	4 (50.0%)	
Left upper lobe	29 (31.6%)	23 (79.3%)	6 (20.7%)	
Left lower lobe	20 (21.7%)	13 (65.0%)	7 (35.0%)	
Size				
Diameter (cm)	1.9 (1.2, 2.8)	2.1 ± 0.9	1.7 (1.0, 3.1)	0.636
The maximum long-axis diameter (cm)	2.3 (1.6, 3.2)	2.4 ± 1.1	2.1 (1.1, 3.4)	0.412
The maximum short-axis diameter (cm)	1.6 (1.0, 2.4)	1.7 ± 0.8	1.5 (0.9, 2.9)	0.907
Density				0.460
Solid	42 (45.7%)	31 (73.8%)	11 (26.2%)	
Part-solid	36 (39.1%)	29 (80.6%)	7 (19.4%)	
Pure GGO	14 (15.2%)	9 (64.3%)	5 (35.7%)	
CT morphological characteristics				
Lobulation	80 (87.0%)	63 (78.8%)	17 (21.2%)	0.067
Spiculation	42 (45.7%)	33 (78.6%)	9 (21.4%)	0.468
Bubble lucency & Cavity	51 (55.4%)	39 (76.5%)	12 (23.5%)	0.716
Pleural indentation	70 (76.1%)	53 (75.7%)	17 (24.3%)	0.778
No. of NSCLC lesions				0.753
Single	77 (83.7%)	57 (74.0%)	20 (26.0%)	
Multiple	15 (16.3%)	12 (80.0%)	3 (20.0%)	

Unless otherwise indicated, data in parentheses are percentages; mean data are ± standard deviation; median data are interquartile range (p25–p75)

*EGFR* epidermal growth factor receptor, *NSCLC* non-small cell lung cancer, *GGO* ground-glass opacity

### Quantitative parameters analysis

Table 4 demonstrates the comparison of DLCT quantitative parameters between the two groups in the AP and VP via univariate and multivariate analysis, respectively. Among quantitative parameters in AP, IC(AP), NIC(AP), INW(AP), and ED(AP) of the mutated EGFR group were significantly higher than those of the wild-type EGFR group (all *p* < 0.05). However, there were no significant differences in the Zeff(AP), VMI_(40keV)_(AP), and VMI_(100keV)_(AP) between the two groups (*p* > 0.05). For DLCT quantitative parameters in VP, IC(VP), NIC(VP), INW(VP), Zeff(VP), λ_HU_(VP), and ED(VP) of the mutated EGFR group were significantly higher than those of the wild-type group (all *p* < 0.05), with no significant differences in the VMI_(40keV)_(VP) or VMI_(100keV)_(VP) (*p* > 0.05). Furthermore, the two groups had no significant differences in AEF (*p* = 0.156) or NAEF (*p* = 0.567).

**Table 4:** Quantitative DLCT parameters between the mutated EGFR group and wild-type EGFR group in the arterial and venous phases

Parameter	All (*n* = 92)	Mutated EGFR group (*n* = 69)	Wild-type EGFR group (*n* = 23)	*p*	Multivariate analysis
AP					
IC (mg/mL)	1.58 (1.27, 2.04)	1.77 (1.41, 2.18)	1.31 ± 0.48	< 0.001	
NIC	0.14 (0.12, 0.19)	0.17 (0.13, 0.22)	0.12 (0.10, 0.13)	< 0.001	
INW (mg/mL)	1.59 (1.29, 2.15)	1.89 ± 0.66	1.31 ± 0.49	< 0.001	< 0.001
Zeff	8.50 ± 0.47	8.55 ± 0.44	8.36 ± 0.55	0.095	
λ_HU_ (HU/keV)	1.72 ± 0.98	1.88 ± 1.02	1.24 ± 0.70	0.006	
VMI_40keV_ (HU)	25.35 (−201.70, 155.65)	65.90 (−170.65, 160.25)	7.70 (−436.10, 103.10)	0.118	
VMI_100keV_ (HU)	−93.10 (−299.28, 50.08)	−73.10 (−283.9, 50.70)	−101.80 (−461.20, 45.80)	0.328	
ED (HU)	40.40 (31.61, 53.78)	46.96 ± 17.18	33.42 ± 13.09	< 0.001	
VP					
IC (mg/mL)	1.75 ± 0.55	1.92 ± 0.49	1.25 ± 0.41	< 0.001	
NIC	0.36 ± 0.11	0.39 ± 0.10	0.25 ± 0.07	< 0.001	< 0.001
INW (mg/mL)	1.77 ± 0.57	1.94 ± 0.50	1.25 ± 0.42	< 0.001	0.137
Zeff	8.48 ± 0.37	8.55 ± 0.35	8.26 ± 0.36	0.001	
λ_HU_ (HU/keV)	1.86 (1.02, 2.55)	2.02 (1.57, 2.62)	1.20 ± 0.72	< 0.001	
VMI_40keV_ (HU)	48.05 (−166.13, 174.20)	78.10 (−147.75, 187.15)	−53.60 (−376.10, 113.00)	0.102	
VMI_100keV_ (HU)	−79.85 (−283.73, 54.28)	−68.00 (−280.55, 55.65)	−114.20 (−395.30, 44.60)	0.220	
ED (HU)	45.72 ± 14.37	49.99 ± 12.62	32.90 ± 11.54	< 0.001	
AEF (%)	94.6 (85.6, 112.0)	96.3 ± 21.7	101.4 (89.9, 112.4)	0.156	
NAEF (%)	44.9 (37.1, 53.2)	45.7 ± 13.4	45.2 (36.8, 64.5)	0.567	

Mean data are ± standard deviation; median data are interquartile range (p25, p75)

*DLCT* dual-layer spectral computed tomography, *NSCLC* non-small cell lung cancer, *EGFR* epidermal growth factor receptor, *AP* arterial phase, *VP* venous phase, *IC* iodine concentration, *NIC* normalized iodine concentration, *INW* iodine no water, *Zeff* effective atomic number, *λ*_*HU*_ the slope of the spectral attenuation curve, *VMI* virtual monochromatic image, *ED* enhancement degree, *AEF* arterial enhancement fraction, *NAEF* normalized arterial enhancement fraction

**Fig. 3:**
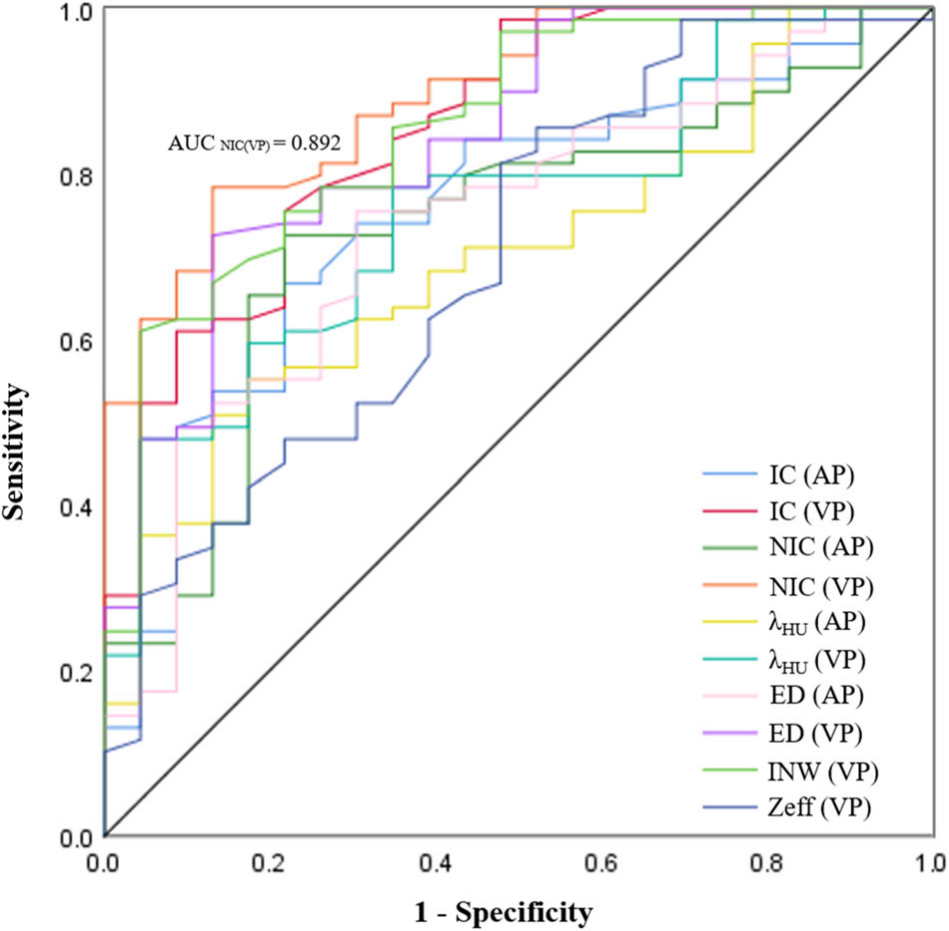
Receiver operating characteristic curves for DLCT quantitative parameters to distinguish EGFR mutation status in NSCLC

Threshold values, accuracy, sensitivity, specificity, and AUC of DLCT quantitative parameters with significant differences between the two groups are shown in Table 5 and Fig. 3. The AUC for determining EGFR mutation status in NSCLC ranged from 0.699 to 0.892. Among those parameters, INW(VP) had the highest diagnostic specificity (95.65%) for identifying EGFR mutation status of patients with NSCLC, followed by NIC(VP) and ED(VP), both with a specificity of 86.96%, and the threshold values were 1.72 mg/mL, 0.30, and 41.95 HU, respectively.

**Table 5:** The diagnostic efficiency of DLCT quantitative parameters between the mutated EGFR group and wild-type EGFR group

Parameter	Threshold	Sensitivity (%)	Specificity (%)	Accuracy (%)	AUC (95% CI)
IC (AP)	1.52	66.67	78.26	69.57	0.755 (0.643, 0.866)
NIC (AP)	0.13	72.46	78.26	76.09	0.734 (0.619, 0.848)
INW (AP)	1.37	81.16	65.22	77.17	0.763 (0.652, 0.874)
λ_HU_ (AP)	1.83	55.07	82.61	61.96	0.699 (0.585, 0.813)
ED (AP)	34.35	75.36	79.57	73.91	0.738 (0.623, 0.852)
IC (VP)	1.52	75.36	78.26	76.09	0.854 (0.765, 0.943)
NIC (VP)	0.30	78.26	86.96	80.44	0.892 (0.823, 0.960)
INW (VP)	1.72	60.87	95.65	69.57	0.855 (0.768, 0.942)
Zeff (VP)	8.23	85.51	47.83	76.09	0.701 (0.575, 0.827)
λ_HU_ (VP)	1.47	78.26	65.22	75.00	0.756 (0.651, 0.862)
ED (VP)	41.95	72.46	86.96	76.09	0.842 (0.750, 0.934)

95% CI means the 95% confidence interval

*AUC* area under the receiver operating characteristic curve, *DLCT* dual-layer spectral computed tomography, *NSCLC* non-small cell lung cancer, *EGFR* epidermal growth factor receptor, *AP* arterial phase, *VP* venous phase, *IC* iodine concentration, *NIC* normalized iodine concentration, *INW* iodine no water, *λ*_*HU*_ the slope of the spectral attenuation curve, *ED* enhancement degree, *Zeff* effective atomic number

**Fig. 4:**
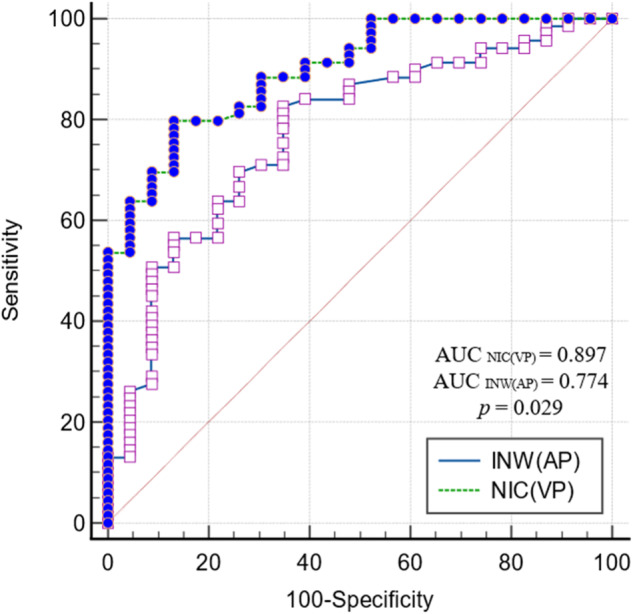
Receiver operating characteristic curves of INW(AP) and NIC(VP) to distinguish EGFR mutation status in NSCLC

Moreover, multivariate logistic regression analyses were performed on the candidate variables mentioned above (age, sex, smoking status, and lobulation) with quantitative parameters that significantly differed between the two groups in the dual-phase scanning of DLCT. The results showed that INW(AP) and NIC(VP) were significant factors for predicting EGFR mutation status, with a sensitivity, a specificity, and accuracy of 82.61%, 65.22%, and 77.17%; and 79.71%, 86.96%, and 81.52%, respectively. The diagnostic efficiency of NIC(VP) (AUC: 0.897, 95% confidence interval [CI]: 0.816–0.951) was significantly higher than that of INW(AP) (AUC: 0.774; 95% CI: 0.675–0.855) for predicting EGFR mutation status in NSCLC (*p* = 0.029; Fig. 4).

## Discussion

The encouraging performance of DLCT quantitative parameters in this study demonstrated that they could provide valid information regarding the EGFR mutation status of NSCLC, with NIC(VP) and INW(AP) identified as key factors. NIC(VP) had the highest predictive efficacy, even higher than that found in previous studies [19, 20].

Previous studies have attempted to detect EGFR mutations in patients with NSCLC using radiomics, specific tumor markers, and morphological features via traditional CT scans [21–23]. The present study found no significant differences in the size, location, density, or morphological features of NSCLC lesions between the two groups, deviating from findings in earlier research [24]. Such inconsistencies could stem from the constrained sample size and potential patient selection bias in this investigation. Clinical features also can provide valuable information about the tumor. The 75.0% EGFR mutation rate among patients with NSCLC and all the mutations being in exons 18 to 21 in this study exceeds the findings of prior reports, likely due to the small sample and the preponderance of early-stage cases compared to the established 40–50% prevalence in Asian lung adenocarcinoma populations [10]. In addition to ethnicity, EGFR mutations in lung cancer are also associated with female sex and non-smoking status [25, 26], consistent with our results. Contrary to prior findings, this study identified no significant difference in sex or smoking history between the two groups [20, 27].

Several malignancies have been noted to show high or abnormal EGFR expression, thereby precipitating sustained activation and amplification of downstream signaling pathways, stimulating physiological and pathological angiogenesis to enhance blood supply to the tumor [28, 29]. Therefore, dynamic contrast-enhanced CT imaging provides additional information about EGFR-mutated NSCLC lesions relative to non-enhanced CT scans. Tacelli et al [30] showed that perfusion CT scanning holds potential as a predictive tool for assessing tumor responsiveness to antiangiogenic therapeutics, yet its utility is limited by variability in patient-specific vascular perfusion characteristics and concerns regarding substantial radiation exposure. The present study showed that ED in the mutated EGFR group was significantly higher than that in the wild-type EGFR group. The VNC images here were obtained by inhibiting iodine in conventional contrast-enhanced CT images. Theoretically, if the quality of the VNC image is good enough, it can replace the true non-contrast image [31], which is of great significance for optimizing the scanning process and reducing the radiation dose.

The present study demonstrated that NIC(VP) exhibited optimal performance in predicting EGFR mutation status. Because iodine is the main component of CT contrast agent, IC can faithfully represent the lesion’s enhancement characteristics, providing a precise evaluation of the angiogenic activity and perfusion status in lung cancer, in contrast to ED. The blood supply of lesions may be increased in NSCLC with EGFR mutations, which could be reflected by IC. This study identified more quantitative parameters that were significantly different between the mutated and wild-type EGFR groups in the VP scans than in the AP scans. The enhanced reliability of the VP scans may be attributable to a more consistent hemodynamic profile in patients, minimizing imaging inconsistencies and yielding more precise quantitative parameters, thereby offering a stable basis for diagnosis. NIC, which calibrates the tumor’s iodine uptake to that of the thoracic aorta, mitigates interpatient hemodynamic variability, enhancing precise EGFR mutation status prediction.

Univariate analysis showed that INW(AP), INW(VP), Zeff(VP), λ_HU_(AP), and λ_HU_(VP) were significantly higher in the EGFR mutated group than in the wild-type EGFR group, although NIC, IC, and ED were not, this is consistent with results of previous studies [19, 20]. INW is indicative of the vascular supply of lung tumors, whereas Zeff reflects the effective atomic number of inorganic constituents within the tumor. Λ_HU_, the slope of the spectral curve obtained by 40 keV–200 keV levels VMI of DLCT, represents the unique linear attenuation coefficient of X-rays by different substances. All three quantitative parameters mentioned above can be used to identify different materials [32, 33], indicating a similar potential to evaluate EGFR mutation status in patients with NSCLC. However, there were no significant differences in VMI_(40keV)_ or VMI_(100keV)_ between the two groups. While low-energy levels of VMI improve lesion delineation, they fail to provide additional diagnostic information beyond that of conventional CT images. Nonetheless, these results need to be further verified through studies with a larger sample size.

Studies have shown quantitative color mapping of the AEF, the ratio of CT value between the AP and the portal venous phase, could increase the diagnostic performance of hepatocellular carcinoma [34]. However, few studies have investigated the clinical value of AEF or NAEF, defined as the ratio of IC or NIC in the arteriovenous phase. For example, AEF could be used to identify and evaluate the function of mediastinal lymph nodes in lung cancer [35, 36]. Wen et al [37] discerned that the NAEF yielded limited utility in discriminating between benign and malignant solid pulmonary nodules. This study marked the inaugural presentation of the NAEF map derived from NIC value; however, it revealed that neither AEF nor NAEF exhibited a significant correlation with EGFR mutation status in NSCLC. Subsequent research with a larger sample size is imperative for validation.

Our study has several limitations. First, it is based on a small sample size from a single-center institution, potentially introducing selection bias. Second, the representation of patients with advanced-stage NSCLC is limited, and investigations into other oncogenic driver mutations, including ALK or KRAS, were not undertaken in this study. Multicenter recruitment is essential to enhance the robustness and generalizability of the findings. Third, the heterogeneity of NSCLC may mean that those quantitative parameters of the two-dimensional spectral DLCT images do not comprehensively represent the biological complexity of the entire tumor, and the potential relevance of quantitative parameters from the tumor’s periphery to the EGFR mutation status was not assessed. Finally, future research should delve into the relationship between the quantitative parameters of DLCT and the efficacy of targeted treatments in lung cancer patients harboring EGFR mutations.

In conclusion, this study demonstrated that quantitative parameters of DLCT were correlated with EGFR mutation status in patients with NSCLC. NIC(VP) might be a potential predictor of EGFR mutation status, which could help to select appropriate and individualized treatment for these patients.

## Data Availability

The datasets used and/or analysed during the current study are available from the corresponding author on reasonable request.

## Abbreviations

AUC: Area under the receiver operating characteristic curve
AEF: Arterial enhancement fraction
AP: Arterial phase
CI: Confidence interval
DLCT: Detector-derived dual-layer spectral CT
DECT: Dual-energy CT
Zeff: Effective atomic number
ED: Enhancement degree
EGFR: Epidermal growth factor receptor
IC: Iodine concentration
INW: Iodine no water
NSCLC: Non-small cell lung cancer
NAEF: Normalized arterial enhancement fraction
NIC: Normalized iodine concentration
ROI: Region of interest
λ_HU_: The slope of the spectral attenuation curve
TKI: Tyrosine kinase inhibitor
VP: Venous phase
VMI: Virtual monochromatic image
VNC: Virtual non-contrast
